# A Pilot Case-Control Study of the Social Media Activity Following Cluster and Non-Cluster Suicides in Australia

**DOI:** 10.3390/ijerph19010343

**Published:** 2021-12-29

**Authors:** Phillip Cheuk Fung Law, Lay San Too, Nicole T. M. Hill, Jo Robinson, Madelyn Gould, Jo-An Occhipinti, Matthew J. Spittal, Katrina Witt, Mark Sinyor, Benedikt Till, Nathaniel Osgood, Ante Prodan, Rifat Zahan, Jane Pirkis

**Affiliations:** 1 Centre for Mental Health, Melbourne School of Population and Global Health, The University of Melbourne, Parkville 3053, Australia; tiffany.too@unimelb.edu.au (L.S.T.); m.spittal@unimelb.edu.au (M.J.S.); j.pirkis@unimelb.edu.au (J.P.); 2 Telethon Kids Institute, Nedlands 6009, Australia; Nicole.Hill@telethonkids.org.au; 3 School of Population and Global Health, The University of Western Australia, Nedlands 6009, Australia; 4 Orygen, Parkville 3052, Australia; jo.robinson@orygen.org.au (J.R.); katrina.witt@orygen.org.au (K.W.); 5 Centre for Youth Mental Health, The University of Melbourne, Parkville 3053, Australia; 6 Departments of Epidemiology and Psychiatry, Columbia University, New York, NY 10032, USA; Madelyn.Gould@nyspi.columbia.edu; 7 Brain and Mind Centre, Faculty of Medicine and Health, University of Sydney, Sydney 2050, Australia; jo-an.occhipinti@sydney.edu.au (J.-A.O.); A.Prodan@westernsydney.edu.au (A.P.); 8 Computer Simulation and Advanced Research Technologies (CSART), Sydney 2021, Australia; 9 Sunnybrook Health Sciences Centre, Department of Psychiatry, University of Toronto, Toronto, ON M4N 3M5, Canada; mark.sinyor@sunnybrook.ca; 10 Unit Suicide Research & Mental Health Promotion, Department of Social and Preventive Medicine, Center for Public Health, Medical University of Vienna, 1090 Vienna, Austria; benedikt.till@meduniwien.ac.at; 11 Department of Computer Science, University of Saskatchewan, Saskatoon, SK S7N 5C9, Canada; nathaniel.osgood@usask.ca (N.O.); rifat.zahan@usask.ca (R.Z.); 12 Translational Health Research Institute, Western Sydney University, Penrith 2751, Australia

**Keywords:** suicide, clusters, contagion, social media

## Abstract

Social media may play a role in the “contagion” mechanism thought to underpin suicide clusters. Our pilot case-control study presented a novel methodological approach to examining whether Facebook activity following cluster and non-cluster suicides differed. We used a scan statistic to identify suicide cluster cases occurring in spatiotemporal clusters and matched each case to 10 non-cluster control suicides. We identified the Facebook accounts of 3/48 cluster cases and 20/480 non-cluster controls and their respective friends-lists and retrieved 48 posthumous posts and replies (text segments) referring to the deceased for the former and 606 for the latter. We examined text segments for “putatively harmful” and “putatively protective” content (e.g., discussion of the suicide method vs. messages discouraging suicidal acts). We also used concept mapping, word-emotion association, and sentiment analysis and gauged user reactions to posts using the reactions-to-posts ratio. We found no “putatively harmful” or “putatively protective” content following any suicides. However, “family” and “son” concepts were more common for cluster cases and “xx”, “sorry” and “loss” concepts were more common for non-cluster controls, and there were twice as many surprise- and disgust-associated words for cluster cases. Posts pertaining to non-cluster controls were four times as receptive as those about cluster cases. We hope that the approach we have presented may help to guide future research to explain suicide clusters and social-media contagion.

## 1. Introduction

Suicide is a major public health problem worldwide [1]. In Australia in 2020, there were 3139 suicides, equating to a rate of 12.2 deaths per 100,000 persons [2]. For every individual who dies by suicide, it has been estimated that an average of 135 people suffer intense grief or are otherwise affected [3].

Sometimes suicides occur in clusters. “Suicide clusters” have traditionally been defined as “groups of suicides that occur closer together in time and space than would normally be expected on the basis of either statistical prediction or community expectation” [4,5]. An estimated 3% of suicides in Australia occur in clusters; 6% of suicides by young people and 2% of those by adults [6]. Although these numbers may not sound high, the impact of suicides that occur in clusters may be particularly devastating for the families, friends and communities of the deceased because of their ripple effect.

The corpus of studies that have been conducted in this area have generally been descriptive, identifying clusters of suicide using statistical techniques [7,8,9,10], or mapping the relationships between members of given clusters [11]. Social links between individuals who died in suicide clusters have been observed in studies of small community settings [12,13] and in nationwide studies [14,15].

To date, relatively few studies have explored the precise mechanism(s) by which suicide clusters may be triggered and perpetuated. One such mechanism is thought to be “contagion” (the social transmission of suicidal behaviour following exposure to the suicide of another) and another is thought to be “assortative relating” (i.e., the co-occurrence of pre-existing risk factors among cluster members that predate the suicide cluster). A recent nationwide study of suicide clusters in Australian youth found evidence of both mechanisms [14], with members of clusters sharing particular suicide methods (suggesting that exposure to the suicide of others may be lead to contagion) and demographic and clinical characteristics (suggesting that cluster members may seek out friends with similar risk profiles, demonstrating assortative relating).

In this pilot study, we have focused particularly on contagion. There is strong evidence from the broader suicide prevention field that the media can play a role in contagion, but the work in relation to media and suicide clusters has been limited. One of the few studies that has explicitly explored this was conducted in the US by Gould et al. [16]. The authors identified suicide clusters, took the first suicide in each cluster, matched these to control non-cluster suicides, and then compared the newspaper reporting that occurred after each. They found that the initial suicides in a cluster were more likely to be followed by high numbers of reports, and by reports that appeared on the newspaper’s front page, included the word “suicide” in the headline, and described the deceased and/or how they died. This suggests that traditional media reporting of suicide may facilitate a contagion effect that might be implicated in youth suicide clusters.

Gould et al.’s [16] study retrospectively examined suicide clusters that occurred between 1988 and 1996, prior to the advent and widespread use of social media. Media effects like those observed by Gould et al. might be expected to be even more striking in social media [11]. Social media is more accessible than traditional media, because the vast majority of people own at least one device that allows them to access social media at any time, and to do so at no or minimal cost. Social media is more pervasive than traditional media, because people spend significant amounts of time communicating via social media, and can share the information en masse with a single click. It is also more interactive and influential, because people create social media content as well as consume it, and content is disseminated among close-knit or diffuse networks.

Not all social media use is potentially harmful in the context of suicide. A growing body of research has focused on how social media can be leveraged to reduce stigma, promote help-seeking, and ultimately prevent suicide [17,18,19]. Nevertheless, there is increasing evidence that social media can influence suicidal behaviour [11,20,21,22] and, in the specific context of suicide clusters, contagion via social media is a significant concern. This is particularly the case given that, despite some developments to incorporate content moderation processes to safeguard users of social media, effective regulations and policies are largely yet to be adopted by social media platforms to maintain online safety, or prevent potential harmful content (e.g., explicit details of a suicide or self-harm event) from being disseminated.

Whether social media confers harm or benefit may depend on the content that is shared through it. We have examined the kind of content that might be described as “putatively harmful” and “putatively protective” [23]. The former might include content that describes a suicide in detail (e.g., providing explicit information about the suicide method) or sensationalises the death, and the latter might include content that presents suicide as undesirable or tragic outcome that can be prevented [23]. We have identified instances in which these two sorts of social media content have been associated with increases and decreases in suicides, respectively [23].

To the best of our knowledge, only one study to date has examined the relationship between suicide cluster related social media and suicidal behaviours [24]. That study involved a survey of 7th to 12th grade students in Stark County in the US state of Ohio during the time when a suicide cluster occurred in the county. It found that exposure to suicide cluster related social media content was associated with a significantly increased odds of suicidal thoughts and behaviours. Students who viewed the content had a higher risk of suicidal ideation but not suicide attempts, whereas students who posted the content had a higher risk of both. However, the study did not examine the nature of the social media content, nor did it explore mechanisms by which social media could lead to suicide clusters. The authors highlight the need to explore how the nature and quality of social media posts may contribute to increased risk of suicidal thoughts and behaviours.

In the present pilot study, we have presented a novel methodological approach for examining the Facebook activity and content that followed the suicide of people whose death formed part of a suicide cluster. We compared this with the Facebook activity and content that followed the suicide of people whose death did not form part of a suicide cluster. Using this approach, we aimed to help clarify the role social media plays in the initiation and perpetuation of suicide clusters. Specifically, we aimed to determine whether there were differences in the linguistic style, social attributes and emotional attributes of Facebook content that followed the two different types of suicide and may contribute to contagion.

## 2. Materials and Methods

### 2.1. Identifying Suicide Clusters

#### 2.1.1. Suicide Data

We used data on deaths due to intentional self-harm obtained from Australia’s National Coronial Information System (NCIS). The NCIS is a national internet-based data storage and retrieval system of coronial records on all reportable deaths [25]. For each death, the system contains information on the deceased (e.g., age, sex, date of birth, residential address) and the circumstances of their death (e.g., date of death, location of death). It also contains reports generated from the investigation into their death, including a police summary of circumstances, an autopsy report, a toxicology report, and the coroner’s findings. We retrieved relevant data from the NCIS on suicides by people of all ages occurring across Australia (except Tasmania, for which ethics approval was not granted) between 2006 and 2017. We excluded suicides with a missing date of death (0.06%) or missing residential address (1.85%). The time period was selected because the dataset covered by it was reliable and complete (more recent data may still be incomplete, because it can two to three years for the investigations into some suicides to be closed).

#### 2.1.2. Population and Geographical Data

Our geographical areas were Statistical Areas Level 2 (SA2s), which are defined by the Australian Statistical Geographical Standards as areas with 3000–25,000 people, designed to represent communities that interact socially and economically, and have a functional centre to which people come to access services [26]. To detect spatiotemporal suicide clusters, we required population estimates and the centroids of the geographical coordinates for all SA2s. Population estimates for all SA2s were obtained from the 2011 census data collected by the Australian Bureau of Statistics (ABS). The centroids of the geographical coordinates for all SA2s were computed using the relevant ABS digital map through ArcGIS software version 10.8.1 (CA, USA).

#### 2.1.3. Scan Analysis

To detect spatiotemporal suicide clusters, we performed the Poisson discrete scan statistic using SaTScan version 9.4.1 [27]. SaTScan is a software that analyses spatial, temporal and space-time point data to identify temporal, spatial, and spatial-temporal clusters. The scan statistic establishes whether clusters exist by running a moving window of a preselected duration over the entire period of interest, seeking evidence of temporal clustering within pre-defined spatial (i.e., geographical) areas. We set the temporal window from a minimum of seven days to a maximum of 15 days because we assumed this is the time when conversations about a suicide are most active, and set the spatial window to commonly used 10% of the population at risk (i.e., the population that is exposed to the risk of suicide) [28]. We chose the typically used circular shape as the shape of the spatial scan window. Monte Carlo simulation [29], a mathematical technique used to conduct quantitative analysis to model risk, was used to identify clusters where the observed number of suicides significantly exceeded the expected number. As with some of our previous analyses [6,10], groups of suicides were considered as “definite clusters” if their *p*-value was lower than 0.05 and “possible clusters” if it was between 0.05 and 0.10. Ultimately, we deemed both to be “clusters”. We chose this approach to account for the statistically rare incidence of suicides. The detection of clusters is somewhat arbitrary, and different approaches will yield different and not necessarily overlapping clusters [15].

### 2.2. Matching Each Cluster Suicide (Cluster Cases) to 10 Non-Cluster Suicides (Non-Cluster Controls)

For each suicide cluster case occurring in a cluster, we randomly selected 10 non-cluster suicides as controls, matching them on sex and age group (≤34, 35−64, and ≥65 years). Non-cluster controls were selected if the individual met either one of the following criteria: (i) they were from a different geographical area (SA2) outside that of the cluster, but within the same state or territory; and (ii) their date of death occurred during a different timeframe within the same year of death as the cluster suicide.

### 2.3. Identifying and Classifying Social Media Data

We examined the Facebook activity and content that followed the cluster and non-cluster suicides. Facebook was our social media platform of choice because it was the most used platform worldwide throughout the majority of our study period (2006–2017), with 2.13 billion monthly users as of the end of 2017 [30].

We identified the public Facebook accounts (including public memorial pages) of those who had died by suicide using their personal information (first name, middle name, last name, sex, date of birth), residential location, and date of death. If a matching account was confirmed—by virtue of no user activity after the date of death and/or an indication that the user had died (e.g., their death had been mentioned or memorialised)—the social media activity from the account and public friends-list accounts associated with it were retrieved. The retrieved posts or replies that referred to the deceased are referred to as text segments. Only text segments written in English within one month after the date of death of the deceased were retrieved for analysis. Prior to analysis, we filtered out all dates and stop words (i.e., common and short function words that were irrelevant to the analysis, e.g., “the”, “is”, “at”, “which”).

We examined text segments for putatively harmful and putatively protective suicide-related content [31]. The putatively harmful content included discussion of the suicide method used by the person who had died (e.g., contained terms commonly associated with suicide and self-harm like “bottle”, “glass”, “hang”, “cut”, “shot”, “jumped”, “bleeding”, “self-inflicted”, “suicide”, “self-harm”), dismissive or hostile responses to individuals expressing suicidal feelings (e.g., “it’s not so bad”, “you need to man up”), or content that was “pro-suicide” (i.e., supporting the idea that a person dies by suicide; e.g., “they did the right thing”). The putatively protective content included messages that discouraged suicidal acts by opposing suicidal behaviour, indicating the negative impacts of suicidal behaviour, or encouraging individuals expressing suicidal feelings to live with statements like “you are not alone, I’m here if you need someone to talk to” and “don’t give up”.

We also conducted concept mapping, word-emotion association analysis, and sentiment analysis. The number of emoji reactions for each Facebook post that referred to the person who had died was also retrieved from each account of cluster and non-cluster suicides and their friends-list accounts. To gauge user reactions to posts, a reactions-to-posts ratio was calculated for each account of cluster and non-cluster suicides by dividing the total number of emoji reactions by the total number of posts that referred to the deceased. The reactions-to-posts ratio did not distinguish between posts made by the same person and posts made by different people.

### 2.4. Comparing Social Media Data

#### 2.4.1. Concept Mapping Using Leximancer

We conducted concept mapping using Leximancer (version 5). Leximancer is a system for extracting semantic content from natural language text to identify concepts and the relationships between them [32]. Concepts are collections of frequently occurring words that appear together in the text and are labelled after those words (e.g., the words “cat” and “dog” frequently co-occur in the text segments that are labelled as “cat” and “dog” concepts). The system uses nonlinear dynamics and machine learning to generate co-occurrence information and a concept map. Concepts are determined by Leximancer as having commonality or contextual similarity if they meet a predefined classification threshold. Semantically similar concepts were manually merged prior to generating concept maps (e.g., “xx”, “xo”, “xoxo”, and “xxx” were merged as “xx”). We compared the concept maps for the text segments for the cluster cases with those for the non-cluster controls. Two-proportions z-tests were conducted in R version 4.0.2 to compare the degree of representation for the non-cluster controls of concepts (i.e., the frequency of occurrence in the text segments of the word after which the concept is labelled) that were widely-represented for the cluster cases, and vice versa.

#### 2.4.2. Word-Emotion Association and Sentiment Analysis

The National Research Council of Canada (NRC; the Government of Canada’s premier organisation for research and development) Word-Emotion Association Lexicon [33,34] of the tidytext package in R version 4.0.2 (Vienna, Austria) was used to identify words associated with negative or positive sentiments and basic emotions (anger, fear, anticipation, trust, surprise, sadness, joy, and disgust). We compared these associations for the cluster cases with those for the non-cluster controls. Two-proportions z-tests were conducted in R version 4.0.2 to compare the degree of representation of sentiments and emotions between the groups.

## 3. Results

### 3.1. Cluster and Non-Cluster Suicides (Cluster Cases and Non-Cluster Controls)

Cluster 1 included 26 people (20 males; age range = 21–68 years; mean age = 44 years) whose suicide occurred between 25 July and 8 August in 2015 (*p* < 0.001). Cluster 2 included 22 people (15 males; age range = 22–90; mean age = 41 years) who died by suicide between 19 December 2015 and 2 January 2016 (*p* = 0.064). A total of 48 suicides were therefore included in the cluster group as cluster cases and these were matched on sex and age group to 480 controls who were included in the non-cluster group.

### 3.2. Facebook Accounts of Cluster Cases and Non-Cluster Controls

We identified the Facebook accounts of three of the 48 cluster cases (6% of cluster cases; all males; age range = 22–49 years; mean age = 36 years), all of whom were from Cluster 2. Their mean number of friends-list accounts was 187. We identified the Facebook accounts of 20 of the 480 non-cluster controls (4% of cluster cases; 15 males; age range = 18–55 years; mean age = 36 years). Their mean number of friends-list accounts was 281.

The first case in the cluster group died on the first day of the cluster (along with three other cluster members who died on the same day), the second died on the second day of the cluster (along with two other cluster members who died on the same day), and the third died on the eleventh day of the cluster (the only member of the cluster who died on that day). Excluding those who died on the same day, the suicide of the first cluster case was followed by 18 suicides, the suicide of the second cluster case was followed by 15 suicides, and the suicide of the third cluster case was followed by nine suicides.

A total of 48 text segments were retrieved from Facebook accounts for the cluster cases. Of these, 46% were posted before the last suicide in the cluster occurred. Thirty of the text segments were from a memorial page for the individual who had died by suicide and 18 were from friends-list accounts. None of the text segments were posted by individuals who subsequently died in the cluster. A total of 606 text segments were retrieved from Facebook accounts for the non-cluster controls, all of which were from friends-list accounts).

### 3.3. Suicide-Related Content

No text segment contained putatively harmful content (i.e., discussed the suicide method or was predominantly “pro-suicide”) for either the cluster cases or non-cluster controls. However, no text segment associated with either group contained putatively protective content (i.e., explicitly discouraging suicidal acts) either.

### 3.4. Concept Map of Social Media Activity

**Figure 1:**
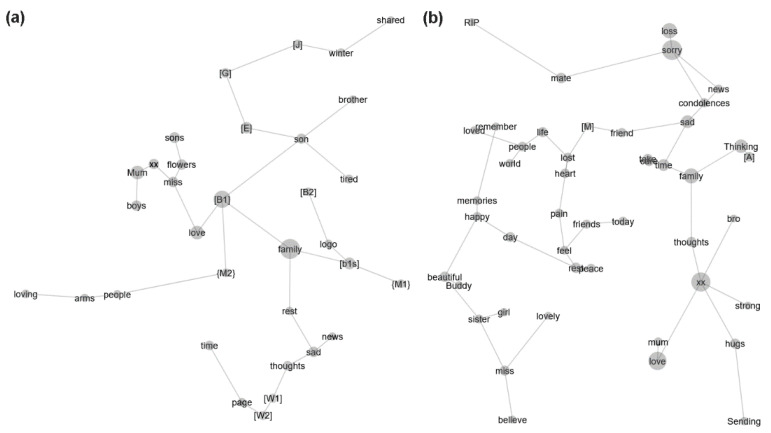
Leximancer concept map for (**a**) the cluster cases and (**b**) the non-cluster controls.

Figure 1 shows the concepts drawn from the individual text segments and describes their relationship to each other via separate concept maps for the cluster cases and non-cluster controls. The labels on each concept map provide the names of concepts that were elicited from the individual text segments. Concept labels that were the names of people and locations were de-identified within “[ ]” and “{ }”, respectively. The size of the circle underlying the concept label reflects the degree of representation of the concept (e.g., the larger a concept’s circle, the more frequently that concept appeared in the text segments). Concepts in closer proximity to each other reflect a greater contextual similarity among the concepts. In each group, the concept map was characterised by individual “branches” comprising distinct concepts—indicating frequent co-occurrence in the same context—and linked by particular concepts with relatively greater representation (present in ≥10% of text segments; discussed below). For the cluster cases, concepts within the branches were relatively more distant from each other, indicating less contextual similarity, and pertained to relatively more names of people and locations. The number of concepts per branch for the cluster cases was also relatively more uniformly distributed, with the number of concepts ranging from a minimum of 1 to a maximum of 6 for the cluster cases, compared with a minimum of 1 to a maximum of 33 concepts for the non-cluster controls).

We only examined the word-like concepts, as concepts pertaining to the names of people and locations do not provide clear, unambiguous insights into the linguistic style, social attributes, and emotional attributes of individual text segments. “Widely-represented” concepts (i.e., present in ≥10% of text segments) for the cluster cases were “family” (present in 35% (17/48) of the text segments), “love” (present in 19% (9/48) of the text segments), and “son” (present in 10% (5/48) of the text segments). The “son” concept for the cluster cases was not observed for the non-cluster controls. Widely-represented concepts for the non-cluster controls were “xx” (an abbreviation for “hugs and kisses”; present in 22% [134/606] of the text segments), “love” (present in 21% (128/606) of the text segments), “sorry” (present in 20% (120/606) of the text segments), and “loss” (present in 11% (69/606) of the text segments). The “sorry” and “loss” concepts for the non-cluster controls were not observed for the cluster cases.

The observed proportion of text segments with the “family” concept was significantly greater for the cluster cases than the non-cluster controls (35% vs. 9% (56/606), respectively; two-proportions z-test, *p* < 0.001). The observed proportion of text segments with the “xx” concept was significantly lower for the cluster cases than for the non-cluster controls (6% (3/48) vs. 22%, respectively; two-proportions z-test, *p* = 0.02). The observed proportion of text segments with the “love” concept was not significantly different between the cluster cases and non-cluster controls (19% vs. 21%, respectively; two-proportions z-test, *p* > 0.05).

### 3.5. Word-Emotion Associations

Approximately twice as many surprise-associated words were mentioned in messages about the cluster cases than the non-cluster controls (10% (12/126) vs. 5% (65/1196), respectively), as were disgust-associated words (4% (5/126) vs. 2% (23/1196), respectively). In contrast, approximately half as many anger-associated words were mentioned in relation to the cluster cases than in relation to the non-cluster controls (5% (6/126) vs. 9% (108/1196), respectively). The cluster cases and non-cluster controls had similar proportions of words associated with trust (21% (27/126) vs. 17% (200/1196), respectively), anticipation (13% (17/126) vs. 14% (163/1196), respectively), fear (9% (11/126) vs. 12% (140/1196), respectively), joy (27% (34/126) vs. 29% (350/1196), respectively), and sadness (11% (14/126) vs. 12% (147/1196), respectively). The observed proportions of words associated with each emotion were not significantly different between the groups (two-proportions z-test, *p* > 0.05).

**Figure 2:**
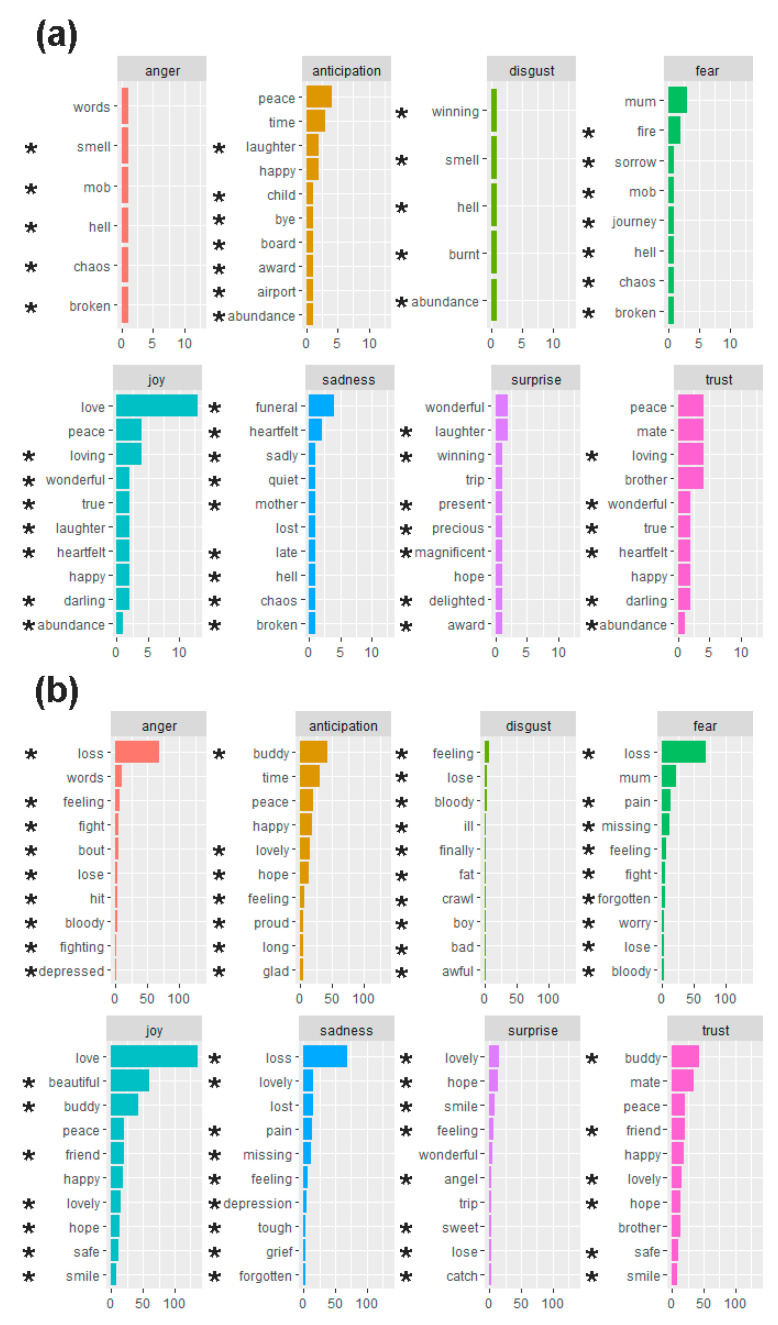
List of the 10 most frequent words associated with each NRC lexicon emotion in (**a**) the cluster cases and (**b**) the non-cluster controls. * Indicates the most frequent words for an emotion for this group that do not fall among the most frequent words for the same emotion in the other group. Words that are not marked with * are common in both groups. The frequency of words is denoted along the *x*-axis.

Figure 2 lists the 10 most frequently used words associated with each emotion in the text segments for the cluster cases and non-cluster controls. We only describe the associations of frequently used words that were exclusive to one group or the other. “Funeral” as a sadness-associated word and “loving” as a trust-associated word were relatively more frequent in relation to the cluster cases than other words of the emotion. “Loss” as an anger-, fear-, and sadness-associated word was markedly most frequently used for the non-cluster controls than other words of these emotions.

### 3.6. Positive- vs. Negative-Sentiment Words

The proportions of positive-sentiment words about cluster cases and non-cluster controls (75% (42/56) vs. 70% (391/557), respectively; two-proportions z-test, *p* > 0.05) were not significantly different and, by extension, nor were the proportions of negative-sentiment words.

**Figure 3:**
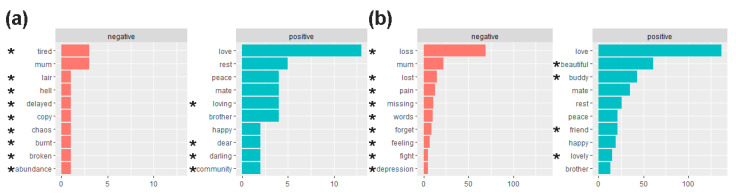
List of the 10 most frequent words associated with NRC lexicon sentiments in (**a**) the cluster cases and (**b**) the non-cluster controls. * the most frequent words for a sentiment for this group that do not fall among the most frequent words for the same sentiment in the other group. Words that are not marked with * are common in both groups. The frequency of words is denoted along the *x*-axis.

Figure 3 lists the 10 most frequently used positive- and negative-sentiment words used in relation to the cluster cases and non-cluster controls. We focus particularly on the sentiments of frequently used words that were exclusive to one group or the other. “Tired” as a negative-sentiment word in the cluster group was relatively more frequent than other negative-sentiment words in that group, and “loss” as a negative-sentiment word in the non-cluster group was more markedly frequent than other negative-sentiment words in that group. Arguably, at least some of the negative-sentiment words in the cluster group (e.g., “hell”, “chaos”, “burnt”) were more intense than most of those in the non-cluster group.

“Loving” as a positive-sentiment word in the cluster group was more frequent than other positive-sentiment words in the group, while “beautiful” and “buddy” as positive-sentiment words in the non-cluster group were more frequent than other positive-sentiment words in the group. There is tentative evidence of positive-sentiment words that may be quite pertinent to the notion of suicide clusters occurring in the cluster group but not in the non-cluster group, like “community”.

### 3.7. Reactions-to-Posts Ratio

Of the 2584 emoji reactions retrieved, one was ‘sad’ (crying face) with the remaining being ‘like’ (thumbs up) and ‘love’ (beating heart). The mean reactions-to-posts ratio of 40.04 in the non-cluster controls was significantly higher than the reactions-to-posts ratio of 8.50 in the cluster cases (Mann–Whitney U Test, *p* < 0.01). This may indicate the reaction to posts in the non-cluster group were at least four times more receptive than for those in the cluster group.

## 4. Discussion

This pilot study presented a methodological approach for examining posthumous social media activity on Facebook in the context of suicide clusters. The approach utilised a scan statistic to identify cluster suicide cases and non-cluster controls and involved concept mapping, word-emotion association, sentiment analysis, and gauging of user reactions. This allowed us to examine the social media activity following suicides that occurred within and outside clusters. We did not identify any “putatively harmful” or “putatively protective” content following the suicides of those who died in a cluster or those who died outside a cluster. However, we did identify some differences in the social media activity that followed the deaths of those in the two groups. The “family” concept was significantly more represented in text segments about cluster cases than non-cluster controls, and the “son” concept, which occurred for the cluster cases was not used in relation to the non-cluster group at all. The “xx” concept (an abbreviation for “hugs and kisses”) was significantly more represented in text segments about the non-cluster controls than the cluster cases, whereas “sorry” and “loss” concepts represented in text segments about the non-cluster controls were not represented in the cluster cases’ text segments at all. We did not find a significant group difference in the proportion of words associated with each emotion (anger, fear, anticipation, trust, surprise, sadness, joy, and disgust) and sentiment (negative and positive). However, we did observe twice as many surprise- and disgust-associated words for the cluster cases, and half as many anger-associated words. Finally, we observed that the reaction to posts pertaining to non-cluster controls was at least four times as receptive as those about those about the cluster cases, as indicated by their reactions-to-posts ratios. The latter finding is unlikely to be explained by difference in the higher average number of friends-list accounts for the non-cluster controls because the magnitude of this difference was much smaller (with the non-cluster controls having about 1.5 times more friends-list accounts than their case counterparts).

It is worth considering what these findings might mean for how people react on social media to suicides occurring in a cluster and how suicide clusters perpetuate. For example, the fact that “family” and “son” concepts were more represented in the cluster group while the “xx”, “sorry”, and “loss” concepts were more represented in the non-cluster group may be informative. The former concepts were explicitly about close, personal, enduring relationships, while the latter were relevant to expressions of condolence following knowledge of the death of the person by suicide. A cautious interpretation of this finding is that individuals responding to non-cluster suicides may not have known the deceased as well as those responding to cluster suicides, and therefore their general expressions of condolence may be a standard reaction to news that someone they didn’t know well has died. However, the finding that the reaction to posts pertaining to non-cluster controls was at least four times as receptive as those about those about the cluster cases may be indicative of relatively stronger social support among the networks of those in the former group. In addition, the seemingly more intense negative-sentiment words in the text segments about the cluster cases (e.g., “chaos”, “burnt”) than in those about the non-cluster controls may be indicative of a more severe negative reaction following knowledge of a suicide occurring in a cluster than following a non-cluster suicide. This is consistent with our findings of a relatively greater proportion of surprise and disgust words relating to the cluster cases, the former of which is in line with reports of a positive association between “surprised” reactions on social media (Twitter) and suicide count [35]. It is also consistent with reports of heightened emotion that may be felt by community members during a cluster [36]. Further detailed work is required to get more insight into these findings before firm conclusions can be drawn.

### 4.1. Limitations

The current pilot study had a number of limitations that must be acknowledged. First, because we only identified two suicide clusters, we made the decision to try to identify the public Facebook accounts of all those who died by suicide in each cluster (and to do the same for the matched non-cluster controls). This is different to the approach taken by Gould, Kleinman [16] who took the first suicide in each cluster and examined the newspaper reporting for them and their matched non-cluster controls. This meant that Gould et al. [16] could be confident that the newspaper reporting about the cluster suicides preceded the other deaths in the cluster, and that they could draw inferences about its likely influence. In our case, we know that the first cluster case was followed by 18 suicides, the second cluster case by 15 suicides, and the third cluster case by nine suicides. We also know that around half of all the social media activity following their deaths occurred before the last suicide in the cluster. This means that we cannot make definitive statements about causality, but we can offer tentative evidence about the kind of social media activity that may occur in the context of a suicide cluster.

Second, despite our best efforts, we were only able to find and confirm public Facebook accounts for a limited number of individuals who died by suicide (three cluster cases [6%] and 20 non-cluster controls [4%]). This meant that the Facebook activity we identified from their public friends-list accounts may not have been representative of either the cluster or non-cluster groups. It also meant that the total number of text segments we had available to us was relatively small, which resulted in issues of power to detect significant differences between the two groups.

Third, our sample covered a broad age range (and thus developmental stages), with cluster cases and non-cluster controls spanning 22–49 years and 18–55 years, respectively. We know from other studies that significant clustering of suicides is most commonly observed in young adults under the age of 25 years [37,38]. Young adults may also be particularly susceptible to contagion effects brought about by media messaging about suicide [39]. Our wide age ranges meant that the social media activity about those who died by suicide may not have been representative of this important age. Additionally, our criteria for non-cluster controls meant that the date of death of four non-cluster controls occurred during the same timeframe as a cluster, despite being from different geographical areas. For the remaining non-cluster controls, the difference in timeframe with cluster cases meant that it is possible for the seasonality of suicide to be a factor. However, it is beyond the scope of our pilot study to examine the seasonality of suicide, largely due to the limited number of identified accounts of cluster cases and non-cluster controls.

Fourth, our analysis was limited to Facebook. Facebook was the most popular platform throughout the majority of the study period, and it also affords more opportunities to examine text than alternative commonly used platforms like Instagram, Snapchat and Twitter, which have text limits or are more visually based. However, patterns of activity on these other platforms may be different, and Facebook is not the preferred platform of all users. In particular, it may not be the platform of choice for young people, whose suicides may be especially susceptible to contagion effects [39,40,41,42]. In addition, we were limited to publicly available posts and replies on Facebook, and it is possible that many conversations about suicide occur via non-public messages and are not posted publicly. These may only be shared with a person’s ‘friends’ but may still be viewable by significant numbers of people. These might be qualitatively different (e.g., expressing private emotions, responding with private information exclusively for the recipient, perhaps containing less socially acceptable language and attitudes). We would argue that public messages have greater reach, and may therefore be particularly influential, although private content transmitted to more select groups of users may be powerful too.

Fifth, we were not able to systematically establish the relationships between a given person who died by suicide and the various individuals in their friends-list, nor the relationships between those individuals in the friends-list and other suicides in the cluster. The relationship is obviously important because it will influence the type of message that an individual might post, for whom, and its potential impact on the recipient. This sort of information would have helped us to interpret our results. For example, family members may be more likely to post messages that are directed towards the person who has died, whereas friends may be more likely to express condolences to the family. Similarly, someone who dies by suicide in a cluster suicide may have been exposed to posthumous social media content about other suicides in the cluster.

Sixth, the text segments pertaining to the cluster cases came from a mix of memorial pages and friends-list accounts, whereas all of the text segments relating to the non-cluster controls came from friends-list accounts. The memorial pages had nearly twice as many text segments as friends-list accounts. A possible explanation is that once a memorial page is created, a notification is (automatically or manually) sent to friends of the person who is being memorialised, thus attracting more posts and replies. We considered excluding the information from the memorial pages and including only that from the friends-list accounts in the comparison, but this would have left us with only 18 text segments for the cluster cases. In any case, we felt that the inclusion of the memorial pages was justified on the grounds that they might be particularly influential in terms of contagion. We know that memorial sites of various forms are not uncommon [43], but further work is required to determine whether those who die by suicide in clusters may be more likely to be memorialised, and whether memorial pages contain qualitatively different content from individual messages on friends-list accounts.

Finally, the concept mapping and the word-emotion association and sentiment analysis might be regarded as fairly blunt approaches to analysing content. For example, the widely-represented “family” concept identified in the concept mapping could be from a post that either said “she couldn’t get along with her family” or “her family has been so great during all of this, they support each other and will get through it”. The former sets up a social norm that conflicts with family as a pathway to suicide that others might emulate, while the latter might lower the barrier to suicide by reassuring a vulnerable person that their loved ones will be fine after their death. Similarly, in the word-emotion analysis, words are pre-defined as being associated with emotions such as “surprise” and “disgust”, but the context in which these words are used may have a bearing. Ideally, we might have presented examples of the text that contained the individual concepts to help the reader understand the context but decided against it because although the accounts and posts were public, the account users did not give us consent to publish their posts/replies. We felt that including verbatim quotations might create privacy issues and might also enable the individuals who were the subjects of the posts/replies and who had died by suicide to be identified. We did, however, conduct a more fulsome content analysis of the text segments in our initial exploration of putatively harmful and putatively protective suicide suicide-related content [31], although we found no examples of either. We also note that other authors have used concept mapping, word-emotion association, and sentiment analysis in examinations of suicide-related social media content. Ideally, we might have deepened our analysis of text segments such as examining cognitive attributes (e.g., sentence framing, category structures) and differences between languages other than English. However, we felt that such analyses are beyond the scope of our pilot study especially given the previously-mentioned limitations.

### 4.2. Future Directions

This pilot study should be regarded as a small pilot only, but it does provide some suggestive evidence that there may be differences in the social media activity that is associated with suicides that occur in a cluster and suicides that occur in isolation. Further work is needed to more firmly establish the nature and extent of these differences.

We hope that our pilot study may serve to guide future research. Future studies might overcome the small sample size issues by involving data from larger countries or multiple countries, using a greater temporal coverage, to identify a greater number of suicide clusters, perhaps with more homogeneous membership. Ideally, these studies might also consider a broader range of social media platforms. They might also look at automating the processes of identifying people and their network of friends and relatives on social media and extracting relevant messages. We performed both of these processes manually, which was time consuming and labour intensive; with additional resourcing and technological advances, it may be easier to at least partially automate these processes. Maximising the number of individuals who are represented in any study will expand the corpus of text segments pertaining to cluster and non-cluster suicides, allowing more refined analyses to be conducted. These might include expanded content analyses of putatively harmful and putatively protective messaging, or more detailed thematic analyses that draw out the “narratives” inherent in particular messages. They might also involve augmented concept mapping and the word-emotion association and sentiment analysis that allows for “deeper dives” into the context surrounding commonly occurring words. Disentangling content on memorial pages from content on friends-list accounts might also be helpful. Anything that can be done to tease out the relationship between members of a suicide cluster is also likely to take our knowledge forward, as are efforts to determine whether those who died by suicide later in a cluster were exposed to social media content about those who died earlier in the cluster.

Alongside this future research, we should consider the best ways to minimise the adverse effects of any social media activity that might promote or perpetuate suicide clusters. We have developed an intervention known as #chatsafe, which is designed to equip young people to discuss suicide safely in an online environment [18,44]. We have recently modified the intervention for use in communities that have experienced one or more suicides and are concerned that a cluster may be emerging. We have deployed this version of #chatsafe in a number of communities in Australia and New Zealand and we are currently examining the potential impact of this as a cluster mitigation strategy. Other options to minimise the potential harm might include working with social media platforms like Facebook to develop standards on what constitutes safe communication following a suicide and how best they might be able to use their algorithms to deploy helpful content and reduce the spread of harmful content.

## 5. Conclusions

Our pilot study is the first to provide data on the kind of social media activity that surrounds suicides occurring in clusters versus suicides that occur in isolation. It provides preliminary evidence that there may be some differences that may help to explain the concept of contagion that is thought to underpin suicide clusters, but further work is needed.

## Data Availability

The data presented in this study are available from the corresponding author. The data are not publicly available in order to retain the anonymity of the Facebook accounts.
